# Coverage-Balancing User Selection in Mobile Crowd Sensing with Budget Constraint

**DOI:** 10.3390/s19102371

**Published:** 2019-05-23

**Authors:** Yanan Wang, Guodong Sun, Xingjian Ding

**Affiliations:** 1 School of Information Science and Technology, Beijing Forestry University, Beijing 100083, China; wangyanan@bjfu.edu.cn; 2 Department of Computer Science, Renmin University, Beijing 100072, China; dxj@ruc.edu.cn

**Keywords:** mobile crowd sensing, coverage area, coverage balance, user selection

## Abstract

Mobile crowd sensing (MCS) is a new computing paradigm for the internet of things, and it is widely accepted as a powerful means to achieve urban-scale sensing and data collection. In the MCS campaign, the smart mobilephone users can detect their surrounding environments with their on-phone sensors and return the sensing data to the MCS organizer. In this paper, we focus on the coverage-balancing user selection (CBUS) problem with a budget constraint. Solving the CBUS problem aims to select a proper subset of users such that their sensing coverage is as large and balancing as possible, yet without violating the budget specified by the MCS campaign. We first propose a novel coverage balance-based sensing utility model, which effectively captures the joint requirement of the MCS requester for coverage area and coverage balance. We then formally define the CBUS problem under the proposed sensing utility model. Because of the NP-hardness of the CBUS problem, we design a heuristic-based algorithm, called MIA, which tactfully employs the maximum independent set model to determine a preliminary subset of users from all the available users and then adjusts this user subset to improve the budget implementation. MIA also includes a fast approach to calculating the area of the union coverage with any complicated boundaries, which is also applicable to any MCS scenarios that are set up with the coverage area-based sensing utility. The extensive numeric experiments show the efficacy of our designs both in coverage balance and in the total coverage area.

## 1. Introduction

**Figure 1:**
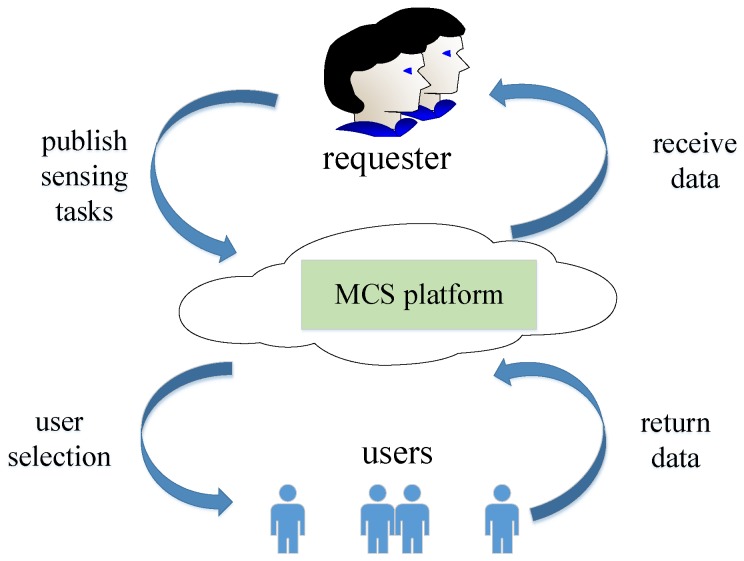
Architecture of typical mobile crowd sensing (MCS) campaigns.

With the proliferation of the smartphones with multiple built-in sensors, recent years have seen more and more mobile crowd sensing (MCS) applications [1]. MCS is a novel technology to achieve urban-scale monitoring and has been applied in traffic monitoring, public safety, and information sharing [2,3,4,5]. A typical MCS campaign, as shown in Figure 1, involves three major components: a requester, an MCS platform, and multiple mobilephone users. The requester publishes some sensing tasks to the MCS platform, in order to obtain some valuable observations on her region of interest (RoI). Since each user can only cover a small fraction of the RoI (i.e., user’s mobilephone is limited in sensing range or coverage), the platform has to recruit multiple users to collaboratively cover the given RoI. Finally, the users who are selected to participate in the MCS campaign return their sensing data to the platform. In general, the requester or the platform needs to pay the selected users for their sensing data.

From the point of view of the requester, the sensing utility offered by the platform depends on how well or in which extent her RoI can be covered [6,7,8,9,10,11]. If her RoI is fully covered, then she can obtain the observation on any spot of her RoI. In real-life MCS scenarios, however, the requester or the platform cannot afford any high charge from the users; in other words, an MCS campaign often comes up with a budget constraint. Thus, one critical issue facing by the MCS user recruitment is to achieve a desirable tradeoff between the sensing utility it can offer to the requester and the payment for users’ sensing data.

A few recent works on MCS user selection have modeled the sensing utility with the sensing coverage and attempted to maximize the sensing utility or minimize the overall cost paid to the selected users. To measure the MCS sensing utility, these previous works usually take into account the total sensing area that the selected users can cover; that is, the larger area those users can cover, the higher sensing utility the requester can obtain. However, such a coverage area-based sensing utility model cannot comprehensively reflect the requester’s requirement—one missing component in evaluating the MCS sensing utility is the geographical balance of sensing coverage that the selected users can offer. In addition to the overall coverage area, we often have to take into account the coverage balance in many typical MCS and wireless sensor network scenarios [12,13,14], especially when the budget is very limited so that the platform only can recruit a handful of users to perform sensing task. For instance, some MCS applications employ compressive sensing or entropy-based approaches to achieve an accurate overall estimation on each sub-area of the given RoI, even with less users who sparsely distribute in the RoI. For such applications, the more balancing the overall sensing coverage, the lower the entropy that the sensing data can lead to, which can also benefit the sparse signal recovery that is highly needed by the compressive sensing. In general, for two MCS user selections that can achieve the same coverage area, the one with the better coverage balance is always more preferable.

In this paper we concentrate on the coverage-balancing user selection (CBUS) problem in the MCS with a budget constraint, and then design effective algorithm to solve this problem. The major contributions of this paper are as follows. To the best of our knowledge, this study is the first work introducing the coverage balance into the MCS sensing utility measurement. We formally define the CBUS problem, which is NP-hard; and we design a heuristic-based algorithm, called MIA, to solve the CBUS problem. MIA also involves a general approach to fast calculating the union sensing coverage with any complicated boundary; this approach could be complementary to solving any user recruitment problems that are defined under the coverage area-based sensing utility model. Finally, we conduct numeric experiments with a wide range of settings, and the results show the efficacy and efficiency of our designs.

The remainder of this paper is structured as follows. Section 2 presents the MCS system model and defines the CBUS problem. Section 3 details the design of MIA. Section 4 compares our designs with two baseline algorithms. Section 5 briefly reviews major research works related to ours. Finally, Section 6 concludes this paper.

## 2. Assumptions and Models

### 2.1. System Model of MCS

We assume that the region of interest (RoI) of requester is a square region of *a* by *a*, and that during the MCS campaign, each user keeps position-fixed or moves only in a very restricted space relative to the RoI, such as in the office room or around the bus stop. In the real-time MCS application, the user movement can be neglected because the sensing tasks published by the requester must be completed in a short term [15]. In the ambient noise and traffic accidents monitoring, for instance, users can complete a sensing task and return sound and photo data in a few seconds or even shorter. Hence it is reasonable to assume stationary users in these real-time MCS scenarios. We denote by U the set of all available users, who are ready to participate in the MCS campaign, and by *A*, the area of requester’s RoI. If user $u_{i}\in U$ is recruited to participate in an MCS campaign, she will charge the platform $c_{i}$, which compensates her efforts in contributing sensing data. We assume that the platform knows the charge of each available user even before the MCS campaign launches.

### 2.2. Models of Sensing Coverage and Sensing Utility

**Figure 2:**
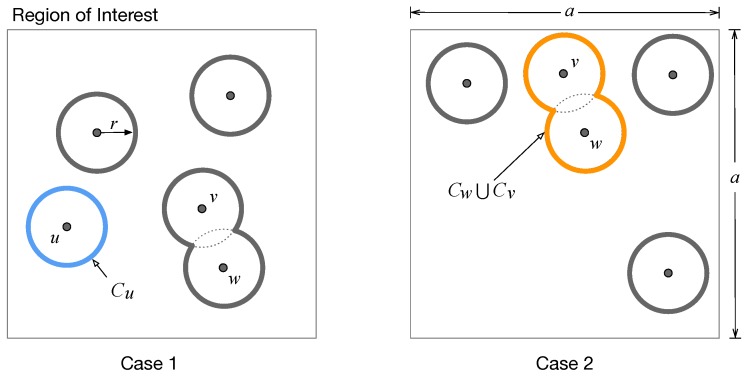
Comparison of two user selection cases, where the left one is more geographically-balancing than the right one, though both can achieve an identical area of total sensing coverage in the square RoI of size a×a.

In this paper, we consider the disk sensing coverage model, as shown in Figure 2; specifically, the on-phone sensor of user *u* can cover a disk-shape region that centers the position of *u* with some sensing radius. We assume that all users have an identical sensing radius *r*, and *r* is much less than *a*. For simplicity, we assume the sensing coverage (disk) of each user is completely within the given RoI. Next we define the sensing utility based on the above sensing coverage model.

**Definition** **1.** *Sensing utility based on coverage area. In an MCS campaign with the user set U and a RoI of area A, the sensing utility that U can achieve is defined by*

(1) $$\phi \left(U\right)=\frac{S({⋃}_{u_{i}\in U}C_{i})}{A},$$

*where $C_{i}$ is the sensing coverage of $u_{i}$ and function S(·) is to calculate the area of the union coverage of all the input users.*

The union coverage of a set of users can be obtained geometrically, but in Section 3.4, we will present a fast approach to effectively estimating the union coverage with any complicated boundary. Clearly, the area-based sensing utility measures how much a RoI can be covered. In Figure 2, for instance, each of the two cases has five users, and both of them achieve the same area-based sensing utility. It is easy to observe in Figure 2 that the total sensing coverage of Case 1 is more balancing than that of Case 2. To offer the requester a better sensing coverage, in this paper, we propose a new metric for profiling the coverage-based sensing utility: this metric jointly considers the area of the total sensing coverage and the geographical balance of the selected users (i.e., their sensing disks). We next present how to quantitatively measure the geographical balance of the users distributing in a RoI.

Let $x_{i}$ represent the 2D coordinate of user $u_{i}$’s position. We first calculate the median or the centroid position $\bar{x}$ by Equation (2), and then, the average distance from any user to position $\bar{x}$ by Equation (3), where d(·) is the function to obtain the Euclidean distance between two positions on plane.

(2) $$\begin{array}{ccc} \bar{x} & = & \frac{1}{n}\sum_{u_{i}\in U}\;x_{i} \end{array}$$

(3) $$\begin{array}{ccc} \bar{d} & = & \sqrt{\frac{1}{n}\sum_{u_{i}\in U}\;d\left(x_{i},\bar{x}\right)} \end{array}$$

**Definition** **2.** *Loss of coverage balance. Given the positions of all users in U and a RoI, the coverage balance loss of U, denoted by ℓ(U), is defined as*

(4) $$\ell \left(U\right)=\frac{\max_{\forall y\in [\mathrm{RoI}]}\min_{u_{i}\in U}\left\{d\left(y,x_{i}\right)\right\}}{\bar{d}},$$

*where [RoI] represents the position set of four RoI corners.*

**Figure 3:**
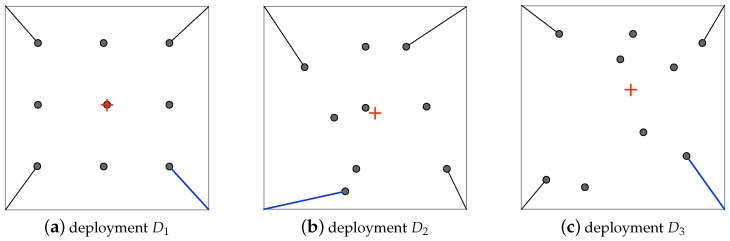
Comparison of three deployments of points in the coverage balance loss. In each deployment, the side length of the square region of interest (RoI) is 10, and the red cross represents the median position of all the nine points. The losses of coverage balance in the three sub-figures are $\ell (D_{1})=2.04$, $\ell (D_{2})=2.23$, and $\ell (D_{3})=2.37$.

**Figure 4:**
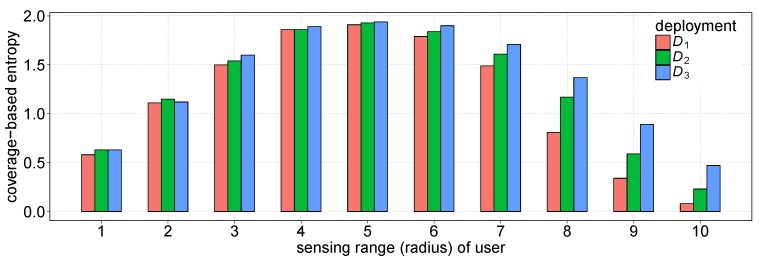
Coverage-based entropies of a random event under the three deployments shown in Figure 3.

The loss of coverage balance defined above measures the geographical distribution of a set of points that are deployed in a 2D plane. Figure 3 shows three deployments of points in an identical square, each of which includes nine points. Intuitively, deployment $D_{1}$ is more balancing than both $D_{2}$ and $D_{3}$. By Definition 2, we can obtain $\ell (D_{1})=2.04$, less than the coverage balance losses of $D_{2}$ and $D_{3}$. Basically, the presented coverage balance loss is established on two deviation measures: (1) the deviation of users from their centroid, i.e., the denominator term of Equation (4), and (2) their deviation from the four RoI corners, i.e., the numerator term of Equation (4). Obviously, the greater the value of ℓ(U), the less balancing the geographical distribution of the users. In the three deployments shown in Figure 3, we calculate the entropy, $H(D_{i})$, for a random event happing within the region of deployment $D_{i}$. We here consider a random event that uniformly occurs in region of $D_{i}$. The entropy for this event is based on the sensing coverage and then it is one effective way of evaluating the sensing utility [16,17]. In detail, let $p_{k}$ be the probability that the random event can be simultaneously detected or covered by *k* users deployed in $D_{i}$, and then we can express the entropy of this event with $H\left(D_{i}\right)=-\sum_{k=1}^{m}p_{k}\log\;p_{k}$, where *m* is the number of users in $D_{i}$ (here in the example of Figure 3, m=9 in each deployment). Figure 4 compares the variations of entropy under three deployments shown in Figure 3. We can see that when the sensing range is small, almost all the users in each deployment cannot cover the random event, i.e., the value of $p_{k}$ is very close to zero, and consequently, the corresponding entropy is low in each deployment. Similarly, larger sensing ranges lead to lower entropy, because every random event can be covered with higher probability. Given a sensing range of user, however, the more balancing the coverage of a deployment, the lower the entropy of a random event, especially when users are densely deployed in RoI. Such an observation indicates that balancing coverage benefits the decrease of sensing uncertainty or the increase of sensing utility. It is necessary to take into account the coverage balance in the MCS user selection.

**Definition** **3.** *Sensing utility based on coverage area and balance (a.k.a coverage balance-based sensing utility). For a given user set U and a RoI, we denote by ν(U) the coverage balance-based sensing utility that U can achieve in this RoI, and it is given by*

(5) $$\nu \left(U\right)=\frac{\phi (U)}{\ell (U)}.$$

Different from the coverage area-based sensing utility model widely used in literature, Definition 3 considers not only the total sensing coverage area of the users but their geographical balance as to the given RoI. Targeting the maximization of such a joint metric, the budget-feasible user recruitment made by the MCS platform can offer the requester a more desirable and beneficial observation about her RoI, especially when the budget is very limited.

### 2.3. Problem Description

In this section, we first formally describe the problem to be addressed in this paper, and then, we analyze its intractability in computation.

**Definition** **4.** *The coverage-balancing user selection (CBUS) problem: to choose a subset U of U such that the coverage balance-based sensing utility of U can be maximized, while the total charge of all the users of U does not violate requester’s budget. The CBUS problem can be formally written as follows.*

(6) $$\begin{array}{cc} \max & \nu (U) \end{array}$$

(7) $$\begin{array}{cc} st. & \;U\subseteq U \end{array}$$

(8) $$\begin{array}{cc} & \;\;\;\;\;\sum_{u_{i}\in U}c_{i}\leq B. \end{array}$$

Since U is finite in size, it must have a finite number of subsets, indicating that there always exists an optimal and nonempty solution for the CBUS problem. Yet the CBUS problem is NP-hard—for a general CBUS instance, we cannot solve it in polynomial time unless P = NP. Theorem 1 proves the computational intractability of the CBUS problem.

**Theorem** **1.** *The general CBUS problem is NP-hard.*

**Proof.**  We first consider a new problem (denoted by r-CBUS) that can be reduced from the CBUS problem and then prove the NP-hardness of r-CBUS. Simply, the r-CBUS problem is selecting a subset of U to maximize the coverage area-based sensing utility, ϕ(U), while the total charge of U is not beyond requester’s budget *B*. By Definition 1, we know ϕ(U)≥0. For any A⊆B⊆U, we have ϕ(A)≤ϕ(B) and we can easily prove the submodularity of function ϕ(·), that is, ϕ(A)+ϕ(B)≥ϕ(A∪B)+ϕ(A∩B). This indicates that the r-CBUS problem is basically a submodular maximization problem with a knapsack constraint, which is a well-known NP-hard problem [18]. In general, the objective function of the CBUS problem, ν(·), is non-monotone over the domain of $2^{U}$, in which the enumerator term ϕ(·) is monotone but the denominator term ℓ(·) is non-monotone. Without considering the non-monotonicity of the CBUS problem, we can reduce CBUS to the r-CBUS problem. Thus we can conclude that the general CBUS problem is at least NP-hard. □

In fact, the non-monotonicity of the objective function ν(·) of CBUS makes it ineffective to solve CBUS directly by employing greedy policy or local search, which are often applied to discrete optimization problems.

## 3. Designs

In this section, we first briefly introduce the basic idea of our algorithm for the CBUS problem and then describe the detailed designs; finally, we show a fast approach to calculating the union coverage, which greatly helps enhance the efficiency of our algorithm in real-life scenarios.

### 3.1. Overview of Our Designs

Obviously, uncontrolled coverage overlaps of multiple users impact the area-based sensing utility. In addition, if most of the selected users are huddled together within a few sub-regions of RoI, we cannot achieve a desirable coverage balance. These observations inspire us to obtain an approximate CBUS solution in such a greedy way: selecting as many users as possible until the budget is violated, while keeping their sensing coverages as separate as possible. With the above heuristic, we design an algorithm, called MIA, which involves two sequential stages.

In the first stage, MIA determines a subset *U* of U such that |U| is maximized while making sure each user of *U* does not overlap with any others. There are three mutually-exclusive situations facing the resulted subset *U*: (1) the total charge of the users of *U* is exactly equal to the budget, (2) *U* overruns the budget, and (3) the budget is not fully fulfilled by *U*, which leaves room for adding extra users into *U*. If one of the last two cases happens, MIA enters the second stage to do the user adjustment: deleting some users from *U* and adding extra users into *U* for the second and the third cases, respectively. The two stages of MIA are described in Section 3.2 and Section 3.3.

### 3.2. Determining Maximum Users without Overlaps

Since we attempt to pick up as many non-overlapping users as possible in the first stage of MIA, we naturally employ the maximum independent set (MIS) problem to model what this stage needs to achieve. For a given undirected, connected graph *G* with *n* vertices and *m* edges, the MIS problem is to insert non-adjacent vertices of *G* into $V_{I}$ such that the size of $V_{I}$ can be maximized [19,20,21]. If an MCS campaign is ready to launch, we can form the users into a disk graph, denoted by $\tilde{G}$, in which the vertices are just the users and an edge is drawn between two vertices if both of them overlap in sensing coverage. With doing so, the MIS solution for $\tilde{G}$ indicates a subset *U* of U such that any two users of *U* are independent or their sensing disks are non-overlapping.

**Figure 5:**
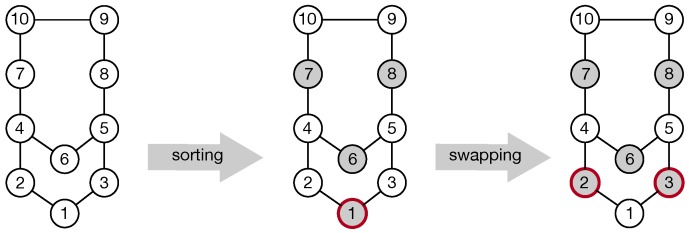
Basic idea of the one-k-swap algorithm in the independent set augmentation. Before and after swapping, the gray vertices form two independent sets with the sizes of four and five, respectively.

The MIS problem is a well-known NP-hard problem in graph theory. One of the state-of-the-art works on MIS is due to Y. Liu, et al. [22], who present the one-k-swap algorithm for general MIS problems. We use this algorithm to satisfy the requirement of the first stage of MIA. To make this paper easy to follow, we next resort to the graph terminology and Figure 5 to briefly explain how the one-k-swap algorithm works with $O(n^{2})$ time complexity.

In essence, one-k-swap is a greedy iterative algorithm, which continues to select qualified vertices and then struggles to improve the results by the swapping operation. Figure 5 shows a walk-through example including 10 vertices. one-k-swap first sorts these vertices in an ascending order of vertex degree, yielding the vertex set $\tilde{V}=\left\{v_{1},v_{2},v_{3},v_{6},v_{7},v_{8},v_{9},v_{10},v_{4},v_{5}\right\}$. Then, one-k-swap sets ${\tilde{V}}_{I}=\emptyset$ and continues to examine the vertices of $\tilde{V}$ one by one, during which it inserts the examined vertex $v_{i}$ into ${\tilde{V}}_{I}$ if and only if $v_{i}$ is non-adjacent with any vertex of ${\tilde{V}}_{I}$; such an iteration terminates with ${\tilde{V}}_{I}=\left\{v_{1},v_{6},v_{7},v_{8}\right\}$, as shown in the middle part of Figure 5. Iteratively, one-k-swap augments ${\tilde{V}}_{I}$ into a larger independent set by continual swaps. More specifically, it exchanges $v_{1}$ with $v_{2}$ and $v_{3}$, i.e., deleting $v_{1}$ from ${\tilde{V}}_{I}$ but adding $v_{2}$ and $v_{3}$ into ${\tilde{V}}_{I}$ in the right part of Figure 5, which increases the size of ${\tilde{V}}_{I}$ by one without producing any conflicts. Such swapping operations stop the moment the size of ${\tilde{V}}_{I}$ does not increase.

As aforementioned, we can form the users of U into a disk graph $\tilde{G}$, according to the overlaps among these users. If we run the one-k-swap algorithm on $\tilde{G}$, we will surely obtain a subset $U_{I}$ of U such that further adding a user into $U_{I}$ will lead to at least one sensing coverage overlap.

### 3.3. Budget-Based User Adjustments

If the determined subset *U* exactly fulfills requester’s budget, i.e., $\sum_{u\in U}c_{u}=B$, we can reasonably terminate MIA; otherwise, we have to do user adjustment on *U* for better budget implementation. The second stage of MIA employs two greedy algorithms, Algorithms 1,2, to deal with the budget overrun and the budget surplus, respectively.

When the budget overrun occurs, i.e., $\sum_{u\in U_{I}}c_{u}>B$, Algorithm 1 greedily deletes users until the budget overrun is removed. The greedy criterion is based on the marginal profit in terms of sensing utility we can obtain if deleting a user from $U_{I}$. From line 3 of Algorithm 1, the marginal profit Δν indicates that how much the total sensing utility will be decreased at a unit user cost. In other words, this greedy criterion guides MIA to preferentially delete the user who charges more but leads to less impact on the total sensing utility. If user $u^{*}$ is determined in line 3 of Algorithm 1, she will be deleted from $U_{I}$.

**Algorithm 1:** Deleting Users in the Case of Budget Overrun.

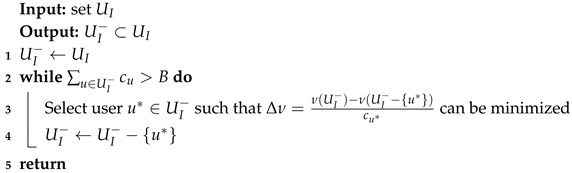



When a budget surplus happens to $U_{I}$, MIA uses Algorithm 2 to add extra users into $U_{I}$, aimed at spending the whole budget. Similar with Algorithm 1, Algorithm 2 also includes a greedy iteration, which always picks out the user from $U\setminus U_{I}$, such that the increase of sensing utility comes with less cost, i.e., the marginal profit Δν in line 3 of Algorithm 2 can be maximized. In the iterations of Algorithm 2, the value of Δν is possibly negative because of the non-decreasing monotonicity of function ν(·). Thus Algorithm 2 can terminate when each available user of $U\setminus U_{I}^{+}$ only contributes non-positive marginal profit.

**Algorithm 2:** Adding Users in the Case of Budget Surplus.

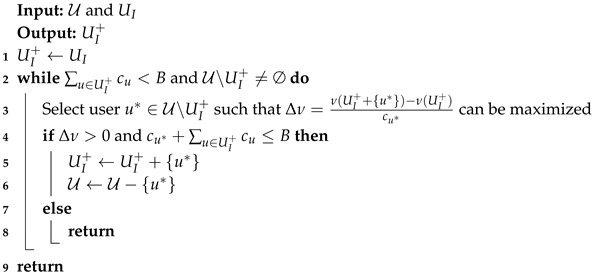



### 3.4. Calculation of Coverage Union

Calculating the sensing coverage area is a major component in the proposed algorithm MIA. For a single user *u*, its coverage area equals $\pi r^{2}$, where *r* is the sensing radius of *u*. For a union sensing coverage of multiple users, furthermore, we can calculate its exact area by the means of geometry computation. For a general union coverage, however, the overlaps formed by the communication disks might be very complicated, resulting in irregular boundary and even non-convex holes; and geometrically calculating the area of such a union coverage will surely lead to intensive geometry computation, which considerably impacts the efficiency of MIA.

In this paper we leverage the grayscale representation and the color aberration to “cut out” the region of the given union coverage, and then, MIA can calculate the corresponding area by pixel counting. Strictly, the accuracy of the above approach depends on the pixel size in use. In practice, however, the pixel sizes are at most in millimeters such that the pixel-based area calculation is adequate in accuracy.

## 4. Evaluation

In this section we conducted numeric experiments to evaluate the performance of our designs. Recall that our main work was to select a subset of users such that the coverage balance-based sensing utility can be maximized under a budget constraint. In our numeric experiments, therefore, we did not need to simulate users’ behaviors in data level as many previous works involving data delivery did in their experiments. Our experiments were conducted with a Java-based homemade simulator, which ran on a PC with a CPU of 2.4 GHz and the memory of 8 GB. We first give the two baseline algorithms, and then describe the experimental setups; finally, we show and analyze the experimental results.

### 4.1. Baseline Algorithms

We compare MIA with two baseline algorithms. The first baseline, called Rand, was selecting users in a completely random way, and it stopped right before the budget overrun occurs. We do not give the details of Rand because it is rather easy to understand. We designed the second baseline algorithm, called the partition-based random selection (ParRand), which was essentially random but employed the RoI partitioning to select users as evenly as possible. ParRand accords with the intuition of making geographically balancing user selection and then serves as a baseline in our evaluation. Algorithm 3 describes the proposed ParRand.

**Algorithm 3:** (ParRand) Partition-Based Random User Selection.

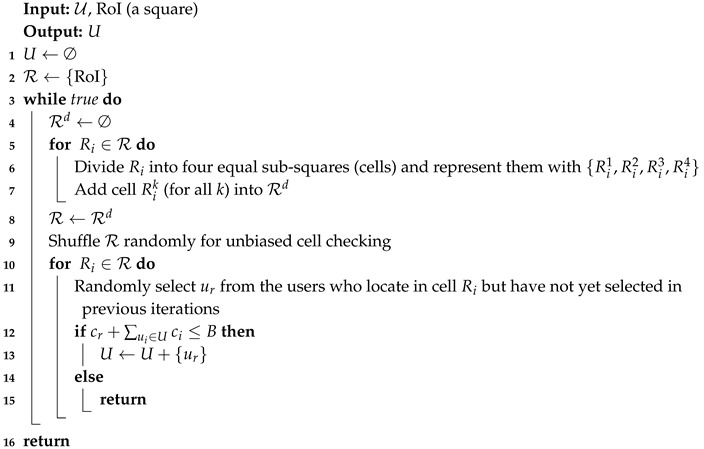



**Figure 6:**
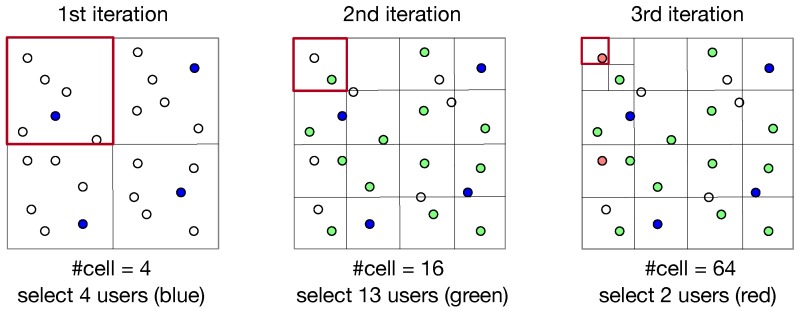
Example of ParRand, which terminates in its third iteration because of the budget overrun.

The main body of ParRand is a while loop. In each iteration ParRand involves two stages: dividing the RoI into multiple equal cells and randomly selecting a user from each cell as long as the budget is not violated. Figure 6 shows an example of ParRand. In the first iteration, ParRand divided the whole RoI into four equal cells (small squares). In line 9 of ParRand, the set R including four cells are shuffled to create a random order of checking every cell of R; such a shuffle leads to unbiased user selection over the current cells. The second stage of the first iteration goes to randomly select four users, each from a cell. If there still is a budget surplus in the first iteration, ParRand will enter the next iteration. In the second iteration, ParRand further divides each cell made by the first iteration into four equal cells (smaller squares) and consequently, there are totally 16 cells in the second iteration. From each of the 16 cells, ParRand attempts to randomly pick out a user who has not yet been selected in previous iterations; in the example of Figure 6, ParRand selects 13 users in its second iteration. Similarly, the third iteration produces 64 cells in the example of Figure 6, but it stops with selecting only two users because the budget overrun will occur otherwise.

In experiments, we found that ParRand performed much better in terms of coverage balance than Rand did. The reason relies on that ParRand always selects users over the evenly-partitioned RoI; on the contrast, Rand selects users directly from the whole RoI. Noticeably, one major disadvantage of ParRand is its computational cost, which is clearly shown by Figure 6. If ParRand terminates with *k* iterations, we then know that ParRand needs to check $4^{i}$ cells in the *i*-th iteration for 1≤i≤k. Thus, *k* iterations of ParRand asymptotically includes $\sum_{1\leq i\leq k}4^{i}=\frac{4}{3}\left(4^{k}-1\right)$ times of cell checking, indicating an exponential growth of time complexity.

### 4.2. Experimental Setup

We set various cases to evaluate our algorithm and compare it with the two baselines in each common case. In all experiments, we set the RoI to be a square with the side length of a=12, and we set the sensing radius of all users to be one. Before each experiment, we uniformly distributed |U| users within the RoI at random, and at the same time, we confined their sensing disks to the RoI. In addition, the charge of each user was determined in each experiment to be a random value with $\left[\overset{ˇ}{c},\hat{c}\right]$, where $\overset{ˇ}{c}$ is always set to one and $\hat{c}$ is a variable to be examined. We repeated 40 experiments with random seeds for each case. Table 1 shows the three experimental cases and the setups of other parameters.

### 4.3. Results and Analyses

**Figure 7:**
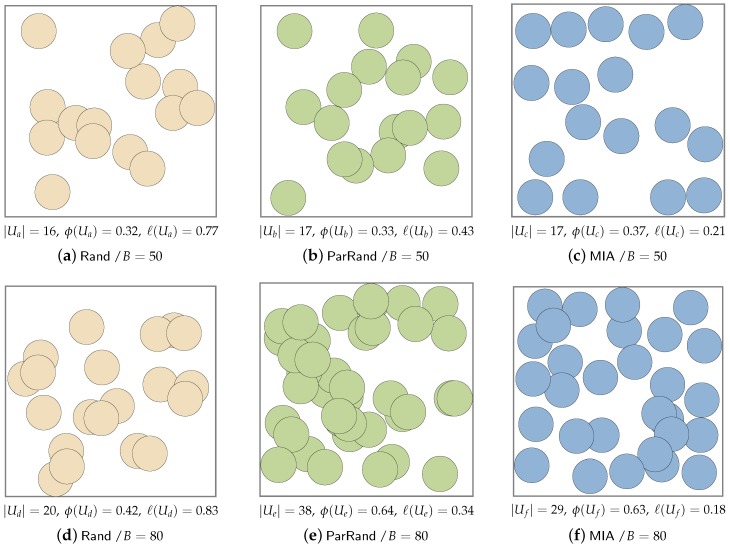
Snapshots of the final sensing coverage after the three algorithms terminate in two common experiments. Both experiments have 200 available users, but one experiment is set with *B* = 50 and the other, with *B* = 80.

Figure 7 shows the overall sensing coverage achieved by the proposed MIA and by the two baselines. These six sub-figures snapshot the experimental results under the setups of 200 users and two different budgets (50 and 80). When *B* is set to 50, only a small fraction of the users can be selected under each algorithm because of the strict budget. By comparing Figure 7a–c, we see that MIA covered more RoI without any coverage overlaps than the other two baselines do, and that MIA achieved better coverage balance. When *B* was set to 80 in Figure 7d–f, all the three algorithms recruit more users than they do with B=50. Rand picked out only 20 users before the budget overrun was hit, significantly less than the users selected by ParRand and MIA. Since Rand selected users without considering their charges, it was easy to saturate the budget. We also find that with a relatively large budget, MIA has no obvious superiority in total sensing coverage than ParRand, and sometimes, ParRand can cover a little more than MIA, as shown in Figure 7e,f. Nevertheless, MIA outperforms ParRand in terms of coverage balance when B=80. MIA achieves a better tradeoff between the coverage balance and the sensing coverage area.

**Figure 8:**
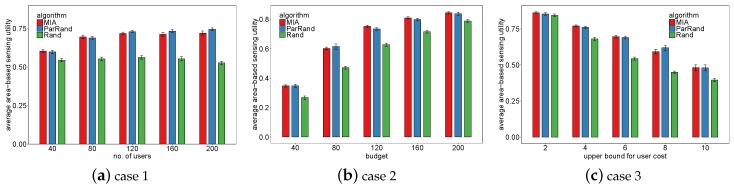
Comparison of the three algorithms in coverage area-based sensing utility.

We evaluate the performance of the three algorithms in coverage area-based sensing utility, and the results are shown in Figure 8. In case 1, Rand always performed worst. With the increase of available users, the total sensing area of MIA was less than, but very close to that of ParRand—the difference was at most 3%. In case 2, which includes 80 available users, the increase of budget incentivizes all the three algorithms to recruit more users. Consequently, when the budget was set to be larger, all of them can cover more and their performance gaps are narrower. In case 3 with a fixed budget of 100, after the upper bound for the user charge goes beyond four, MIA and ParRand obviously perform better than Rand.

**Figure 9:**
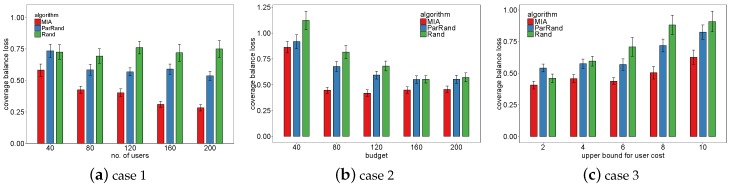
Comparison of the three algorithms in coverage balance loss.

We in Figure 9 compare the three algorithms in terms of coverage balance loss. In Figure 9a, the coverage balance loss increasingly reduces: the loss value drops by half when the number of available users increases from 40 up to 200. Furthermore, Figure 9b,c can also demonstrate the MIA’s distinct advantage in coverage balance over the two baselines. In case 2, the higher the budget, the more users were selected by each of the three algorithms, and thus, their performance in coverage balance is continually improved as the budget increases. Although the increase of the upper bound for the user charge reduces the number of users finally selected by each algorithm, MIA rises more slowly in coverage balance loss than the other two baselines do.

## 5. Related Work

Coverage problem is a critical topic in wireless sensor networks [23], in which the sensing utility is reckoned to be closely related to the sensing coverage. In the community of wireless sensor networks, the coverage problems often target minimizing the number of sensors while guaranteeing a full and connected coverage. In wireless sensor networks, the coverage determination needs to create a connected network among the selected sensors. In MCS, however, the user’s mobilephone can directly connect with the platform via the internet. In addition, the works on wireless sensor network coverage problems put less attention on coverage balance.

In the MCS community, researchers have done some preliminary works in recent years. Wang et al. [7] consider the coverage area-based sensing utility model, and propose an algorithm to minimize the overall system cost with some QoS constraint. Xiong et al. [24] design CrowdTasker for MCS, which aims at maximizing the coverage quality of sensing tasks while agreeing with a given incentive budget constraint. Zhang et al. [8] target the maximization of sensing quality with budget constraint under the MCS scenario different from ours. They measure the sensing quality by the coverage of mobilephone users that can be achieved in user movement; in detail, their coverage-based sensing quality is determined by the number of points of interest that a user can cover. Xiong et al. [9] present iCrowd, which recruits users to cover more subareas of the RoI while minimizing the payment to the selected users. Similar with our coverage model, Obinikpo et al. [25] avoid more than two users who cover the same target, and they propose a scheduling policy to save the energy of sensing devices. But they do not consider the budget constraint in their models. Yu et al. [10] consider the *k*-coverage model, which needs multiple users to cover any given point of interest. Girolami et al. [26] propose the binary coverage model and the aging coverage model for MCS, and employ spatial interpolation strategy to improve the sensing utility. In their models, however, the RoI is divided into a finite set of equal square tiles, and they measure the degree of coverage by the number of the tiles that can be covered by users’ sensing disks. Basically, their models measure the sensing utility based on the sensing area, and do not take into account the coverage balance.

In spite of the ongoing advances in MCS, there is still a lack of effective solutions for the user selection that can achieve balancing coverage under a constrained budget.

## 6. Conclusions

In this paper we have proposed the CBUS problem, which is to achieve the coverage-balancing user selection in the MCS with a budget constraint. We have proven the NP-hardness of CBUS and then designed algorithm MIA to approximately solve it. MIA leverages the maximum independent set model to first obtain a relatively balancing coverage over the given RoI, and then includes two algorithms to deal with the budget overrun and the budget surplus. Extensive numeric experiments show the efficacy of our designs both in the coverage balance and in the total coverage area. In the future work, we will extend our model of coverage balance and design effective algorithm such that they can profile the MCS user selection in a spatiotemporal way, and then, can well improve the sensing utility of long-term sensing tasks.

## Figures and Tables

**Table 1:** Setup of parameters and variable ranges used in each case.

Parameter	Description	Value or Range		
		Case 1	Case 2	Case 3
|U|	the number of initial users	40∼200 (step = 40)	80	80
*B*	the budget	100	40∼200 (step = 40)	100
$\hat{c}$	the upper bound for user cost	6	6	2∼10 (step = 2)
